# Short-term anabolic agent and sequential therapy to improve bone mineral density and bone turnover markers in patients with osteoporotic hip fractures

**DOI:** 10.1186/s13018-025-06084-5

**Published:** 2025-07-16

**Authors:** Jun Young Park, Jun-Young Lim, Tae Kang Kim, Byung Woo Cho, Hyuck Min Kwon, Kwan Kyu Park, Woo-Suk Lee

**Affiliations:** 1 Department of Orthopaedic surgery, Yongin Severance Hospital, Yonsei University College of Medicine, 363 Dongbaekjukjeon-daero, Giheung-gu, Seoul, Yongin-si, 16995 Gyeonggi-do Republic of Korea; 2 Department of Orthopaedic Surgery, Severance Hospital, Yonsei University College of Medicine, Seoul, Republic of Korea; 3 Yonsei University Wonju College of Medicine, Wonju, Republic of Korea; 4 Department of Orthopaedic Surgery, Gangnam Severance Hospital, Yonsei University College of Medicine, Seoul, Republic of Korea

**Keywords:** Hip Fracture, Sequential Therapy, Teriparatide, Romosozumab, Denosumab, Bone Mineral Density, Bone Turnover Markers

## Abstract

**Background:**

Therapy using anabolic and antiresorptive agents in sequence is reportedly effective for severe osteoporosis management. However, evidence for this approach in osteoporotic hip fracture patients remains limited. This study aimed to evaluate the effectiveness of sequential therapy using short-term anabolic agents followed by antiresorptive treatment on bone mineral density (BMD) and bone turnover markers (BTMs) in patients with osteoporotic hip fractures.

**Methods:**

We retrospectively reviewed 330 patients with osteoporotic hip fractures between February 2022 and December 2023 and selected 113 patients. The patients were categorized into a sequential group (*n* = 68), who received an anabolic agent (teriparatide or romosozumab) for three to six months, followed by two doses of denosumab administered at six-month intervals, and a non-sequential group (*n* = 45), who received anabolic agent monotherapy. The primary outcome was mean change in BMD at the lumbar spine (LS), femoral neck (FN), and total hip (TH) at one-year postoperatively. Secondary outcomes were the osteoporosis medication profile and mean change of 25-hydroxyvitamin D₃ (25(OH)D₃) and BTMs including C-terminal telopeptide (CTX) and procollagen type 1 N-terminal propeptide (P1NP).

**Results:**

The sequential group showed significant increases in LS-, FN-, and TH-BMD at one-year follow-up (3.6 ± 3.7%, 4.4 ± 7.9%, and 1.9 ± 4.1%, respectively; *p* < 0.001 for all). In contrast, the non-sequential group showed non-significant changes in BMD at all sites. In the sequential group, CTX levels decreased significantly (0.57 ± 0.39 to 0.32 ± 0.30 ng/ml, *p* < 0.001), whereas the non-sequential group showed a non-significant increase in CTX levels (0.73 ± 0.47 to 0.90 ± 0.56 ng/ml, *p* = 0.44). P1NP levels decreased significantly in the sequential group (88.2 ± 65.7 to 66.2 ± 62.8 µg/L, *p* < 0.001). The 25(OH)D₃ levels improved in both groups but were higher in the sequential group (20.7 ± 11.1 to 37.2 ± 13.6 ng/mL).

**Conclusion:**

Sequential therapy with short-term anabolic agents followed by antiresorptive therapy significantly improved BMD and normalized BTMs in patients with osteoporotic hip fractures. This treatment approach may be an effective strategy to enhance bone health and potentially reduce subsequent fracture risk in this high-risk population.

## Introduction

Osteoporotic hip fractures represent one of the most serious consequences of osteoporosis and are associated with high morbidity, mortality, and healthcare costs. The one-year mortality rate following hip fracture can reach 20–30%, and fewer than half of survivors regain their pre-fracture functional status [1–3]. Furthermore, patients who suffer a hip fracture have a 2.5-fold increased risk of subsequent fractures [4, 5]. Therefore, effective osteoporosis management following hip fracture is crucial for both fracture healing and prevention of future fractures [6, 7].

Although antiresorptive agents have traditionally been the basis of treatment, these agents primarily prevent further bone loss rather than rebuild diminished bone architecture [8, 9]. Their use may be insufficient for patients with severe osteoporosis or established fractures [10–12]. Anabolic agents have demonstrated superior efficacy in increasing bone mineral density (BMD) and reducing fracture risk compared to antiresorptive agents [13]. However, the bone-forming effects of anabolic therapy diminish over time, and discontinuation without follow-up antiresorptive therapy leads to rapid loss of gained BMD [14]. Consequently, the concept of sequential therapy has emerged, in which anabolic treatment is followed by antiresorptive agents to maintain and potentially enhance gains in bone mass and strength [15, 16].

Evidence from recent studies supports the efficacy of sequential therapy. Meta-analyses have demonstrated that sequential therapy, starting with anabolic agents and then antiresorptive drugs, provides significant reduction in fracture risk and improved BMD outcomes [15, 17, 18]. Research has shown that anabolic agents such as teriparatide have particularly strong effects on trabecular bone structure, while also providing benefits for cortical bone volume [19]. When considering implementation, analyses in cost-effectiveness have suggested that sequential therapy approaches must be balanced with economic considerations, particularly in healthcare systems with limited resources [20].

Current clinical guidelines recommend anabolic agents as first-line therapy for very-high-risk patients, including those with recent hip fractures, followed by antiresorptive therapy [21]. However, real-world implementation of sequential therapy in patients with hip fractures remains limited, and the optimal duration of anabolic therapy before transitioning to antiresorptive agents is uncertain. While most studies have employed 18 to 24 months of anabolic therapy [22, 23], shorter durations might be more feasible in a real-world setting, particularly for elderly patients.

Given the above background, this study aimed to evaluate the effectiveness of sequential therapy using short-term anabolic agents followed by antiresorptive treatment on BMD and bone turnover markers (BTMs) in patients with osteoporotic hip fractures. We hypothesized that this approach would result in greater improvements in BMD and more favorable changes in BTMs compared to non-sequential therapy.

## Materials and methods

### Study design and patient selection

This retrospective cohort study was conducted at a regional university hospital. The study protocol was approved by the Institutional Review Board of our Institution (IRB No. 9-2025-0038). The requirement for informed consent was waived due to the retrospective nature of the study.

We reviewed the medical records of patients who underwent surgery for osteoporotic hip fractures from February 2022 to December 2023. Osteoporotic hip fractures were defined as fragility fractures of the proximal femur occurring from a fall from standing height or lower. The inclusion criteria were patients who underwent surgical treatment (arthroplasty or internal fixation) for osteoporotic hip fracture between February 2022 and December 2023. The exclusion criteria were (1) age younger than 55 years, (2) pathologic fractures or atypical femoral fractures, (3) infection or other abnormal findings, (4) incomplete or unavailable BMD measurements or BTM evaluations, (5) death within one year or loss to follow-up, (6) osteoporosis treatment non-initiated and (7) concurrent use of anabolic and antiresorptive agents without sequencing.

**Fig. 1:**
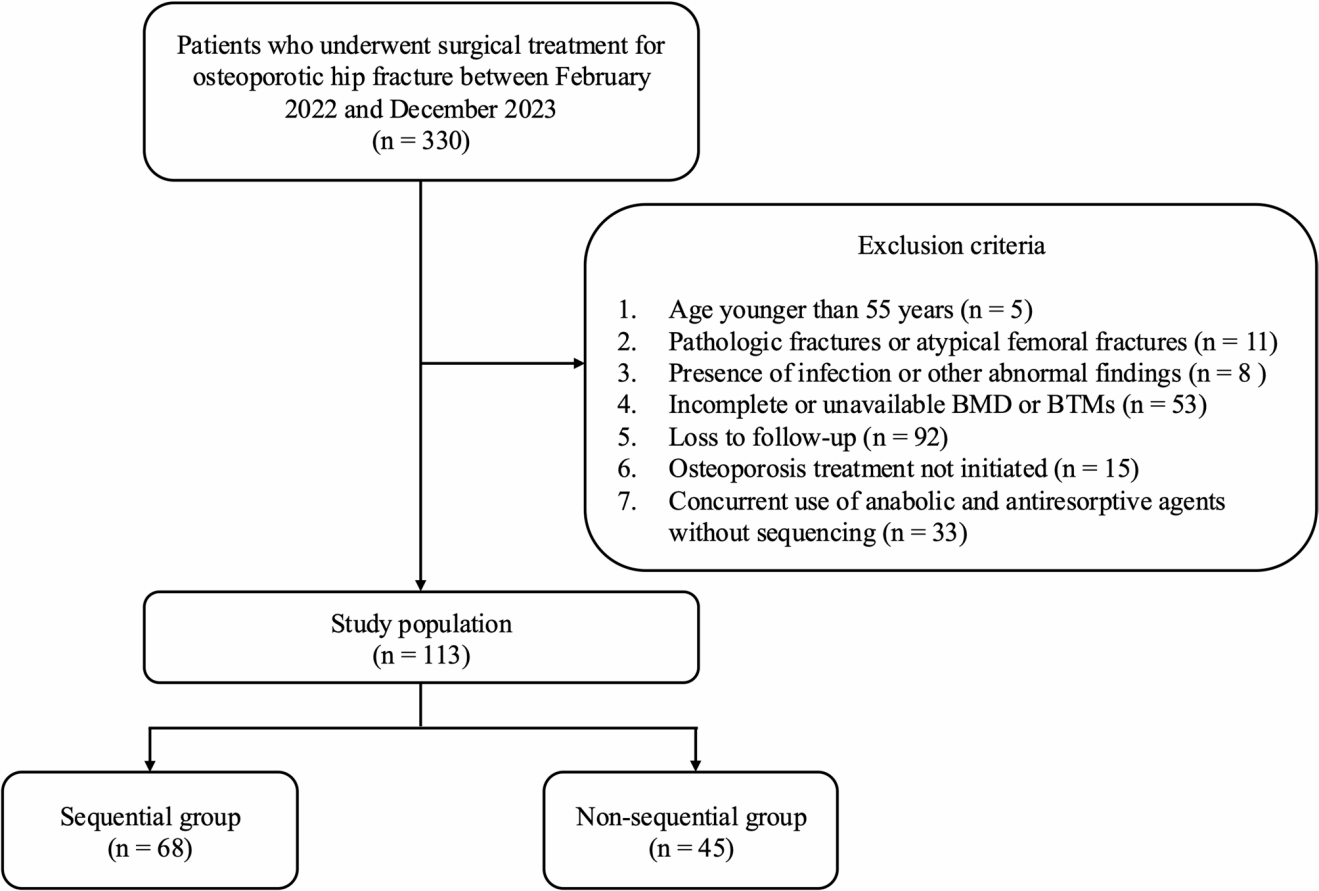
CONSORT flow diagram of patient selection

After applying the above criteria, 113 of the initial 330 patients remained and were categorized into two groups (Figure 1). The sequential therapy group (*n* = 68) was defined as the patients who received teriparatide or romosozumab for three to six months and then denosumab twice, once at six months and once at 12 months. The non-sequential group (*n* = 45) included the patients who received anabolic agent monotherapy.

### Treatment protocol

In the sequential group, 60 patients (88.2%) received teriparatide (20 µg daily subcutaneous injection) and eight patients (11.8%) received romosozumab (210 mg monthly subcutaneous injection) for three to six months, followed by denosumab (60 mg subcutaneous injection given twice over the next six months). In the non-sequential group, all 45 patients received single-shot therapy (anabolic agent alone). All patients, except one with heart failure, were supplemented daily with calcium (1,000 mg) and vitamin D (800 IU) throughout the study period.

### Outcome measurements

BMD was measured at the lumbar spine (LS), femoral neck (FN), and total hip (TH) using dual-energy X-ray absorptiometry (DXA, Lunar iDXA, GE Healthcare) at baseline (preoperative) and 12 months after surgery. BMD was expressed in g/cm². The least significant change (LSC), representing the 95% confidence level for a true biological change in BMD, was set at 0.02 g/cm² for the LS- and FN-BMD and 0.015 g/cm² for the TH-BMD, based on previous studies [24].

BTMs were measured at baseline (preoperative) and at 12 months following the operation. Procollagen type 1 N-terminal propeptide (P1NP) was measured as a bone-formation marker, and C-terminal telopeptide of type I collagen (CTX) was measured as a bone resorption marker. Additionally, serum 25-hydroxyvitamin D₃ (25(OH)D₃) levels were measured at the same time points.

### Statistical analysis

Continuous variables are presented as mean ± standard deviation, and categorical variables are presented as numbers and percentages. Baseline characteristics were compared between the sequential and non-sequential groups using independent t-tests for continuous variables and chi-square or Fisher’s exact tests for categorical variables. Changes in BMD and BTMs within each group were analyzed using paired t-tests and Wilcoxon signed-rank test depending on the normality of the data. Between-group differences were analyzed using independent t-tests. The proportion of patients achieving clinically significant BMD improvement (defined as an increase ≥ 0.015 g/cm²) was compared using chi-square tests. A p-value < 0.05 was considered statistically significant. All statistical analyses were conducted using R software, version 4.4.0.

## Results

The baseline demographic and clinical characteristics of the study population are presented in Table 1. The mean age was 81.4 ± 6.8 years, and 83.2% were female. No significant differences existed between the sequential and non-sequential groups in terms of age, sex distribution, body mass index, Charlson comorbidity index, American Society of Anesthesiologists classification, fracture type, surgery type, or baseline BMD (all *p* > 0.05). Intertrochanteric fractures were the most common type (54.0%), followed by femoral neck fractures (46.0%). Surgical treatments included bipolar hemiarthroplasty in 50 patients (44.2%), internal fixation using a plate and screw construct in two patients (1.8%), and intramedullary nailing in 61 patients (54.0%).

**Table 1:** Demographic and clinical characteristics of hip fracture patients: comparison between sequential and non-sequential treatment groups

	Sequential group	Non-sequential group	Total	*p*
**N**,** hips**	68	45	113	
**Age**	81.9 ± 5.8	80.7 ± 8.2	81.4 ± 6.8	0.42
**Female**	60 (88.2%)	34 (75.6%)	94 (83.2%)	0.13
**Body mass index**	23.1 ± 3.8	22.3 ± 3.5	22.8 ± 3.7	0.22
**CCI**	4.6 ± 0.8	4.9 ± 1.4	4.7 ± 1.1	0.18
**ASA classification**				0.28
**2**	12 (17.7%)	11 (24.4%)	23 (20.4%)	
**3**	56 (82.3%)	33 (73.3%)	89 (78.8%)	
**4**	0 ( 0.0%)	1 (2.2%)	1 (0.9%)	
**Type of fracture**				0.43
**Femur neck**	30 (44.1%)	22 (48.9%)	52 (46.0%)	
**Intertrochanteric**	38 (55.9%)	23 (51.1%)	61 (54.0%)	
**Type of operation**				0.85
**Bipolar hemiarthroplasty**	29 (42.6%)	21 (46.7%)	50 (44.2%)	
**CRIF c plate**	1 (1.5%)	1 (2.2%)	2 (1.8%)	
**CRIF c IM nail**	38 (55.9%)	23 (51.1%)	61 (54.0%)	
**Operated side (Rt/Lt)**	35/33	27/18	62/51	0.44
**Time to surgery**	4.4 ± 5.2	4.2 ± 3.5	4.4 ± 4.6	0.79
**Baseline BMD**,** T-score**				
**Lumbar spine**	-1.7 ± 1.1	-1.6 ± 1.4	-1.7 ± 1.2	0.66
**Femoral neck**	-2.8 ± 0.7	-2.7 ± 0.9	-2.8 ± 0.8	0.45
**Total hip**	-1.8 ± 0.8	-1.8 ± 1.1	-1.8 ± 0.9	0.97

CCI, Charlson comorbidity index; ASA, American Society of Anesthesiologists; CRIF, closed reduction and internal fixation; IM, intramedullary; Rt, right; Lt, left; BMD, bone mineral density

As shown in Table 2, all patients in the sequential group (100%) received sequential therapy, whereas the non-sequential group received anabolic agent monotherapy (100%). In the sequential group, 88.2% received teriparatide, 11.8% received romosozumab, and 100% received denosumab. In the non-sequential group, 84.4% received teriparatide and 15.6% received romosozumab. The cumulative exposure to osteoporosis medications was significantly greater in the sequential group for teriparatide (17.1 ± 5.3 vs. 4.1 ± 2.0 weeks, *p* < 0.001) and romosozumab (19.0 ± 3.6 vs. 3.9 ± 3.5 weeks, *p* < 0.001) based on treatment duration. The non-sequential group received no denosumab injections.

**Table 2:** Treatment strategies and osteoporosis medication profiles in sequential and non-sequential groups

	Sequential group	Non-sequential group
**Type of osteoporosis treatment**		
**Sequential**	68	0
**Single shot**	0	45
**Type of osteoporosis agent**		
**Teriparatide**	60	38
**Romosozumab**	8	7
**Denosumab**	68	0
**Treatment duration of anabolic agents**,** weeks**		
**Teriparatide**	17.1 ± 5.3	4.1 ± 2.0
**Romosozumab**	19.0 ± 3.6	3.9 ± 3.5
**Total**	17.3 ± 5.1	4.0 ± 2.1
**Cumulative numbers of antiresoprtive agent injections**		
**Denosumab**	2.0 ± 0.0	0

**Fig. 2:**
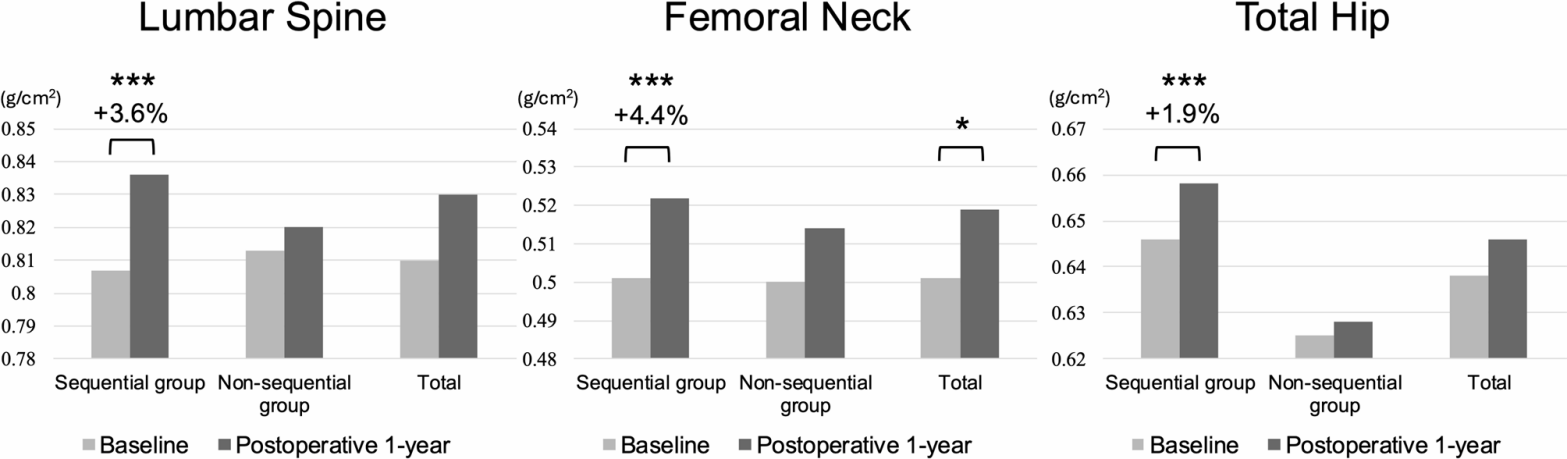
Changes in bone mineral density at the lumbar spine, femoral neck, and total hip at postoperative 1-year

The BMD changes after one year of treatment are summarized in Table 3 (Figure 2). The sequential group showed significant increases in BMD at all measured sites: LS-BMD (3.6 ± 3.7%, 0.807 ± 0.123 to 0.836 ± 0.129 g/cm², *p* < 0.001), FN-BMD (4.4 ± 7.9%, 0.501 ± 0.080 to 0.522 ± 0.088 g/cm², *p* < 0.001), and TH-BMD (1.9 ± 4.1%, 0.646 ± 0.099 to 0.658 ± 0.101 g/cm², *p* < 0.001). In contrast, the non-sequential group showed non-significant changes in BMD at all sites: LS-BMD (1.0 ± 3.9%, 0.813 ± 0.163 to 0.820 ± 0.163 g/cm², *p* = 0.11), FN-BMD (1.7 ± 7.6%, 0.500 ± 0.096 to 0.514 ± 0.113 g/cm², *p* = 0.32), and TH-BMD (0.4 ± 3.2%, 0.625 ± 0.139 to 0.628 ± 0.143 g/cm², *p* = 0.41). The proportion of patients achieving clinically significant BMD improvement above the LSC was significantly higher in the sequential group compared to the non-sequential group for LS-BMD (69.1% vs. 33.3%) and FN-BMD (69.1% vs. 28.9%). For TH-BMD, the difference was smaller (32.4% vs. 28.9%).

**Table 3:** Comparison of bone mineral density changes after one year of treatment between sequential and non-sequential groups: (1) lumbar spine, (2) femoral neck, and (3) total hip

	Lumbar spine BMD				Femoral neck BMD				Total hip BMD				
	**Baseline** (**g/cm²)**	**Postop 1-year** (**g/cm²)**	**% Increase in BMD**	***p***	**Baseline** (**g/cm²)**	**Postop 1-year** (**g/cm²)**	**% Increase in BMD**	***p***	**Baseline** (**g/cm²)**	**Postop 1-year** (**g/cm²)**		**% Increase in BMD**	***p***
**Sequential group**	0.807 ± 0.123	0.836 ± 0.129	3.6 ± 3.7	< 0.001	0.501 ± 0.080	0.522 ± 0.088	4.4 ± 7.9	< 0.001	0.646 ± 0.099	0.658 ± 0.101	1.9 ± 4.1		< 0.001
**Non-sequential group**	0.813 ± 0.163	0.820 ± 0.163	1.0 ± 3.9	0.11	0.500 ± 0.096	0.514 ± 0.113	1.7 ± 7.6	0.32	0.625 ± 0.139	0.628 ± 0.143	0.4 ± 3.2		0.41
**Total**	0.810 ± 0.139	0.830 ± 0.143	2.5 ± 4.0	0.08	0.501 ± 0.086	0.519 ± 0.098	3.4 ± 7.9	0.01	0.638 ± 0.116	0.646 ± 0.119	1.3 ± 3.9		0.51

*p*-values represent within-group comparisons (baseline vs. postoperative 1 year)

BMD, bone mineral density; Postop; postoperative

**Fig. 3:**
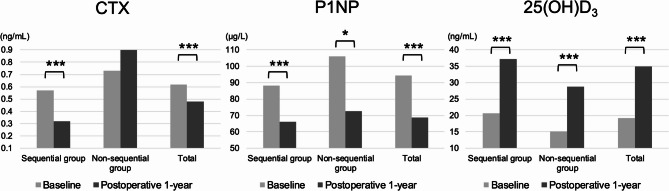
Changes in bone turnover markers and serum 25(oh)d₃ levels after postoperative 1-year CTx, C-terminal telopeptide; P1NP, Procollagen type 1 N-terminal propeptide; 25(OH)D, 25-hydroxyvitamin D₃

The changes in BTMs are presented in Table 4 (Figure 3). In the sequential group, CTX levels decreased significantly from baseline (0.57 ± 0.39 ng/ml) to one year (0.32 ± 0.30 ng/ml, *p* < 0.001), indicating reduced bone resorption. In contrast, the non-sequential group showed a non-significant increase in CTX levels from baseline (0.73 ± 0.47 ng/ml) to one year (0.90 ± 0.56 ng/ml, *p* = 0.44). P1NP levels decreased significantly in the sequential group from baseline (88.2 ± 65.7 ng/ml) to one year (66.2 ± 62.8 ng/ml, *p* < 0.001). In the non-sequential group, P1NP also significantly decreased from baseline (106.2 ± 97.4 ng/ml) to one year (72.7 ± 76.8 ng/ml) (*p* < 0.05). Serum 25(OH) vitamin D_3_ levels increased in both groups, with the sequential group showing higher values at both baseline (20.7 ± 11.1 vs. 15.2 ± 9.6 ng/ml) and one year (37.2 ± 13.6 vs. 28.7 ± 16.6 ng/ml, *p* < 0.001 for both groups).

**Table 4:** Comparison of vitamin D and bone turnover markers between sequential and non-sequential groups at baseline and one year Follow-up

	CTX			P1NP			25(OH)D₃			
	**Baseline** (**ng/mL)**	**Postop 1-year** (**ng/mL)**	***p***	**Baseline** (**µg/L)**	**Postop 1-year** (**µg/L)**	***p***	**Baseline** (**ng/mL)**	**Postop 1-year (ng/mL)**	***p***	
**Sequential group**	0.57 ± 0.39	0.32 ± 0.30	< 0.001	88.2 ± 65.7	66.2 ± 62.8	< 0.001	20.7 ± 11.1	37.2 ± 13.6	< 0.001	
**Non-sequential group**	0.73 ± 0.47	0.90 ± 0.56	0.44	106.2 ± 97.4	72.7 ± 76.8	0.01	15.2 ± 9.6	28.7 ± 16.6	< 0.001	
**Total**	0.62 ± 0.42	0.48 ± 0.47	< 0.001	94.5 ± 78.4	68.7 ± 67.6	< 0.001	19.2 ± 10.9	34.9 ± 14.8	< 0.001	

*p*-values represent within-group comparisons (baseline vs. postoperative 1 year)

CTx, C-terminal telopeptide; P1NP, procollagen type 1 N-terminal propeptide; 25(OH)D₃, 25-hydroxyvitamin D₃; Postop, postoperative

## Discussion

This study that sequential therapy using short-term anabolic agents followed by antiresorptive therapy significantly improves BMD and favorably alters BTMs in patients with osteoporotic hip fractures. The sequential group showed significant increases in BMD at all measured sites, with the most pronounced effect at FN-BMD (4.4%). In contrast, the non-sequential group showed minimal and non-significant changes in BMD.

These findings underscore the importance of appropriate osteoporosis management in the early post-operative period following hip fracture surgery, particularly in terms of proper medication sequencing for these high-risk patients. The concept of sequential therapy is based on the understanding that anabolic agents rapidly stimulate new bone formation, creating a larger bone surface area for subsequent antiresorptive therapy [25]. This approach maximizes the therapeutic effects of both medication classes and can lead to greater BMD gains than either agent alone or in reverse sequence [26].

The significant improvement in FN-BMD (4.4%) in our sequential group is particularly noteworthy as the femoral neck is predominantly composed of cortical bone, which typically responds more slowly to osteoporosis therapy than trabecular bone [27]. This finding suggests that even short-term anabolic therapy followed by antiresorptive treatment can effectively improve BMD in cortical bone, which is crucial for reducing the risk of subsequent hip fractures.

Regarding site-specific effects, Takahashi et al. demonstrated through quantitative computed tomography that daily teriparatide had a particularly strong effect on trabecular bone of the vertebra (50.8% increase in BMDs after 18 months) [19]. Although daily teriparatide did not significantly increase cortical BMD of the proximal femur, it did increase cortical bone volume, suggesting that structural improvements occur even without substantial increases in mineral density. Our findings of significant BMD improvement at both trabecular-rich lumbar and cortical-rich femoral sites with short-term anabolic therapy suggest that even abbreviated exposure to these agents may initiate important structural changes that are maintained with subsequent antiresorptive therapy.

The changes in BTMs provide additional insights into the mechanism of action of short-term sequential therapy [28, 29]. The significant decrease in CTX levels in the sequential group indicates effective suppression of bone resorption, while the relative maintenance of P1NP levels suggests ongoing bone formation. This pattern is consistent with what Tsai et al. described as the “anabolic window,” where bone formation exceeds bone resorption, leading to a net gain in bone mass [30]. Our findings suggest that even abbreviated anabolic therapy can create a sufficient anabolic window that can be preserved with prompt transition to antiresorptive therapy.

Our study utilized a relatively short duration of anabolic therapy (mean 17.3 ± 5.1 weeks) before transitioning to antiresorptive treatment. While our results align with those from the DATA-Switch study, our approach used a significantly shorter duration of anabolic therapy compared to most previous studies and guidelines, which typically recommend 12 to 24 months [22, 23]. These findings have important clinical implications for management of osteoporosis in patients with hip fractures. Despite clear guidelines recommending aggressive osteoporosis treatment after hip fracture, substantial barriers to implementation exist in real-world practice [31].

Several studies have documented the challenges of maintaining patients on full-length anabolic agent courses. Hagino et al. reported that up to 40% of patients discontinued teriparatide within 12 months owing to cost concerns, injection fatigue, side effects, or comorbidities [32]. Similarly, a claims database analysis by Modi et al. found that adherence to anabolic therapy decreased substantially after six months, with fewer than 30% of patients completing the full 18–24 month course [33]. Financial constraints, patient tolerance, healthcare system limitations, and reimbursement policies often hinder the recommended 12 to 24 months of anabolic therapy. These challenges are often magnified in elderly hip fracture patients, who frequently have multiple comorbidities, polypharmacy issues, and limited financial resources [34–36].

Our study provides evidence that abbreviated anabolic therapy followed by antiresorptive agents can significantly improve BMD and bone turnover markers, which may lower the barrier to implementing sequential therapy in this population. This pragmatic sequential approach recognizes the realities of clinical practice while providing patients with many of the benefits of sequential therapy. By demonstrating that even short-term anabolic therapy produces meaningful benefits when followed by appropriate antiresorptive treatment, our findings may encourage more widespread adoption of this therapeutic strategy in high-risk patients for whom the standard duration of anabolic therapy is not feasible.

In a complementary study, Park et al. evaluated the impact of sequential therapy using short-term teriparatide followed by denosumab compared with denosumab alone in patients with osteoporotic hip fractures [37]. They found that, while both approaches improved spine BMD, only the sequential approach showed a trend toward improved FN-BMD. Their findings, which were consistent with our results, suggest that sequential therapy provides advantages for improving bone strength at the hip—a crucial factor for preventing subsequent fractures.

Additionally, our study highlighted the high prevalence of vitamin D deficiency in this patient population, with baseline levels below 20 ng/ml in both groups. Despite supplementation, many patients had suboptimal levels at one year, emphasizing the need for more aggressive vitamin D repletion in elderly patients with hip fractures. This finding aligns with recent research suggesting that vitamin D status can influence the efficacy of osteoporosis medications, particularly anabolic agents [37].

From an economic perspective, the abbreviated sequential approach we studied might represent a more cost-effective option than standard-duration anabolic therapy. While formal cost-effectiveness analyses were beyond the scope of our study, the significantly reduced duration of the expensive anabolic phase (3–6 months versus 12–24 months) could substantially lower treatment costs while maintaining significant clinical benefits [21]. This economic advantage, coupled with the clinical benefits we observed, indicates the abbreviated sequential approach as an attractive option for healthcare systems with limited resources.

Based on our findings, we recommend that future clinical practice should emphasize the continuation of sequential therapy with at least two doses of denosumab following short-term anabolic therapy. The significant improvements in BMD and BTMs observed in our sequential therapy group were achieved through this specific protocol of short-term anabolic agents followed by two denosumab injections administered at six-month intervals. This approach ensures sustained antiresorptive effect while maintaining the gains achieved during the anabolic phase [38]. Future research should focus on identifying the optimal number and timing of denosumab injections to maximize treatment efficacy following short-term anabolic therapy, as well as evaluating long-term outcomes through extended follow-up in this high-risk population.

Our study had several limitations. First, as a retrospective study, it was subject to selection bias. Although we used propensity score matching to minimize bias, and the baseline characteristics were similar between groups, unmeasured confounders might have influenced the results. Second, we did not collect data on prior use of antiresorptive agents. Considering the DATA-Switch study finding that BMD can decrease when teriparatide is administered after denosumab, this could have introduced bias in our results. Third, the follow-up period of one year might have been insufficient to fully capture the long-term effects of sequential therapy on fracture risk. Fourth, the study was conducted at a single center, which might have limited the generalizability of the findings. Fifth, we did not perform a detailed analysis of fracture healing parameters or functional outcomes, which would have provided a more comprehensive assessment of treatment benefits in this population. Finally, we did not confirm whether our laboratory results directly translate into secondary fracture prevention. We believe that future prospective studies are necessary to investigate the clinical significance of sequential therapy in terms of actual fracture prevention.

## Conclusion

Sequential therapy with short-term anabolic agents (mean 17.3 ± 5.1 weeks) followed by antiresorptive therapy significantly improved BMD and normalized BTMs in patients following osteoporotic hip fracture surgery. This abbreviated sequential approach resulted in greater BMD gains, particularly at the FN-BMD (4.4%), and more favorable BTM profiles compared to non-sequential therapy. These findings support the proposed pragmatic sequential therapy as a cost-effective strategy that balances clinical efficacy with real-world constraints, potentially improving medication adherence in elderly hip fracture patients with multiple comorbidities. Further prospective studies with longer follow-up periods are warranted to evaluate the impact on subsequent fracture risk and to determine the optimal minimal duration of anabolic therapy.

## Data Availability

No datasets were generated or analysed during the current study.
